# Trapping and destruction of blood-borne syngeneic leukaemia cells in lung, liver and spleen of normal and leukaemic rats.

**DOI:** 10.1038/bjc.1976.82

**Published:** 1976-05

**Authors:** T. E. Sadler, P. Alexander

## Abstract

Leukaemic cells from rats with a lymphoid (HRL) or myeloid (SAL) leukaemia were labelled with 125IUDR and injected i.v. into either normal or leukaemic syngeneic recipients. The fate of the injected cells was studied in terms of the radioactivity in various tissues at various times up to 24 h later. In normal animals the leukaemia cells were destroyed rapidly in the reticulo-endothelial (RE) system; immediately after injection most recoverable activity was in the lung, with smaller amounts in the blood, spleen and liver but by 24 h only 20-30% of the injected activity could be recovered. In leukaemic recipients with high numbers of blasts in the blood the amount of activity recoverable from the lungs and bone-marrow was markedly reduced, while that in the blood was doubled. Nonetheless, the overall rate at which radioactivity was eliminated was not significantly different from that found in normal rats, in spite of the fact that the RE system was extensively infiltrated by leukaemia cells.


*Br. J. Cancer* (1976) **33**, 512

### TRAPPING AND DESTRUCTION OF BLOOD-BORNE SYNGENEIC LEUKAEMIA CELLS IN LUNG, LIVER AND SPLEEN OF NORMAL AND LEUKAEMIC RATS

T. E. SADLER* AND P. ALEXANDER

*From the Division of Tumour Immunology, Chester Beatty Research Institute, Sutton, Surrey*

Received 15 December 1975 Accepted 21 January 1976

**Summary.—Leukaemic cells from rats with a lymphoid (HRL) or myeloid (SAL) leukaemia were labelled with $^{125}$IUDR and injected i.v. into either normal or leukaemic syngeneic recipients. The fate of the injected cells was studied in terms of the radioactivity in various tissues at various times up to 24 h later.**

**In normal animals the leukaemia cells were destroyed rapidly in the reticulo-endothelial (RE) system; immediately after injection most recoverable activity was in the lung, with smaller amounts in the blood, spleen and liver but by 24 h only 20–30% of the injected activity could be recovered.**

**In leukaemic recipients with high numbers of blasts in the blood the amount of activity recoverable from the lungs and bone-marrow was markedly reduced, while that in the blood was doubled. Nonetheless, the overall rate at which radio-activity was eliminated was not significantly different from that found in normal rats, in spite of the fact that the RE system was extensively infiltrated by leukaemia cells.**

SEVERAL studies have shown that when radio-labelled viable tumour cells are injected intravenously (i.v.) into syngeneic recipients the great majority are trapped and killed in the lung, liver and spleen (Selecki, 1959; Hofer, Prensky and Hughes, 1969; Fidler, 1970, 1973). The fraction of cells which survives injection by the i.v. route and causes tumours is very small and less than that found for cells given intraperitoneally or subcutaneously (Hofer *et al.*, 1969). The majority of tumour cells trapped in the lung lyse within a few hours and if radio-labelled in their DNA the label appears in the urine in the form of low molecular weight substances. The mechanism for this rapid destruction of tumour cells in the lung is not known, but it seems unlikely that an immunological reaction of the host directed against tumour-specific surface antigens can be involved, as the rate of destruction is modified only slightly by immunosuppression or prior immunization (van den Brenk *et al.*, 1975; Weiss, Glaves and Waite, 1974). The extent and rate at which labelled i.v.-inoculated tumour cells are killed by the reticuloendothelial (RE) system is very much greater than that of similarly injected radio-labelled lymphocytes (Shorter and Bollman, 1960; Woodruff and Gesner, 1969). This raises the possibility that the RE system may have a means of discriminating between malignant and non-malignant cells. Indeed, macrophages that have been activated by, for example, endotoxin, kill lymphoma and sarcoma cells *in vitro* (Alexander and Evans, 1971; Hibbs, Lambert and Remington, 1972). There is evidence that this mechanism contributes to the *in vivo* destruction of some tumours by endotoxin (Parr, Wheeler

* Present address: Department of Surgery, Royal Postgraduate Medical School, Ducane Road, London, W12 0H8.

and Alexander, 1973) and other agents that render macrophages non-specifically cytotoxic. In particular, the increased resistance of the lungs to i.v. challenge with tumour cells which is observed when rodents are treated with *Corynebacterium parvum* (Proctor, Rudenstam and Alexander, 1973; van Putten *et al.*, 1975) may be attributed to increased tumouricidal potential of lung macrophages.

The concept that the RE system, and in particular the lung, recognizes malignant cells through a characteristic surface property implies that such a mechanism is impaired when there are cells circulating in the blood as in leukaemic animals. To test this hypothesis, the fate and rate of death of i.v.-inoculated leukaemia cells were studied in rats, using two transplantable acute leukaemias which arose spontaneously. The HRL leukaemia is an acute lymphoblastic leukaemia and the SAL an acute myelogenous leukaemia (Wrathmell and Alexander, 1973; Wrathmell, 1976). The leukaemia cells were labelled *in vitro* and *in vivo* with the DNA precursor, $^{125}$I-5-iodo-2′ deoxyuridine ($^{125}$IUDR) and injected i.v. into normal and leukaemic syngeneic recipients.

### MATERIALS AND METHODS

*Rats.*—Pure line, barrier-maintained, male hooded and female August rats, 8–10 weeks old, were taken from our own colony as required.

*Leukaemias: HRL.*—A lymphoid leukaemia (HRL) (Wrathmell, 1976), which arose spontaneously in a male hooded rat in 1971, was maintained by blood passage of $5 \times 10^6$ spleen or blood cells every 14 days. Frozen stock cells were referred to every 30–35 weeks.

*SAL.*—A myeloid leukaemia (SAL) (Wrathmell, 1976), which arose spontaneously in a female August rat in 1968, was maintained by blood passage of $10^5$ spleen or blood cells every 7 days. A stock of frozen cells was referred to every 15–20 weeks.

*Collection of cells.*—Leukaemic rats were bled by cardiac puncture using a heparinized syringe. The blood was centrifuged for 5 min at 25,000 *g* and the buffy coat resuspended in TC 199 (Wellcome). These cells were centrifuged for 5 min at 240 *g* and resuspended at a concentration of $10^7$ cells/ml. 65–85% blast cells were present depending on the leukaemic state of the donor.

*Radiolabelling and injection of cells:* $^{125}$*IUDR* in vitro.—Cells were routinely labelled with $^{125}$IUDR (Radiochemical Centre, Amersham, sp. act. 1–6 $\mu$C/mg) (Hughes *et al.*, 1964; Fidler, 1970). They were incubated for 1 h with 0·1 $\mu$C/ml at 38°C, washed 3 times centrifuging for 5 min at 240 *g* and finally suspended at concentrations of $10^8$ cells/ml (Hall and Smith, 1970). A cell viability of 95% was usual. Incorporation of $^{125}$IUDR into the DNA of the two cell types differed. Activity taken up by the HRL cells was only one tenth that of the SAL cells. Autoradiographs of buffy coat smears were used to determine the percentage of blasts labelled: 27% of the HRL blasts and 68% of the SAL blasts were labelled. A crude nucleic acid extraction of the labelled cells with trichloracetic acid (Schneider, 1945) indicated that approximately 75% of the activity associated with the leukaemia cells was attached to the DNA.

Rats were injected whilst under light ether anaesthesia with 0·5 ml of tissue culture fluid containing $5 \times 10^7$ cells in the lateral tail vein.

$^{125}$*IUDR* in vivo.—Thirteen days after the inoculation of leukaemia cells, hooded rats were given 150 $\mu$C $^{125}$IUDR i.v. at 0 and 8 h. The rats were exsanguinated at 24 h and 0·5 ml volumes of blood were immediately injected into recipient rats.

$^{3}$*H-thymidine* in vitro.—When tissue autoradiographs were required, $^{3}$H-thymidine (Radiochemical Centre, Amersham, specific activity 23 Ci/mmol) was used as the label. The cells were treated as for *in vitro* $^{125}$IUDR-labelling except that they were incubated with 1 $\mu$C/ml $^{3}$H-thymidine for 1 h at 38°C (Hall and Smith, 1970).

*Heat-killed cells.*—Labelled cells were killed by heating in a 55°C water bath for 45 min. They were washed before transfusion.

*Collection of material for radioassay and counting procedures.*—Recipient rats were killed in groups of 5 at various intervals from nominally zero time (1–5 min) to 24 h after injection of $^{125}$IUDR-labelled cells. In every case the following tissues were removed: lung, liver, spleen, small gut (flushed of contents), thymus, lymph nodes (mesenteric, mediastinal, superficial and deep cervical), bone marrow (as femur), and blood taken by cardiac puncture. In a few cases the whole rat was dissected. The tissues were cleared of connective tissue and fat, washed in normal saline and blotted dry before weighing and placing in a counting vial. Blood was divided into 2 samples, one left whole, the other centrifuged and the serum removed for counting. The radioactivity in each vial was counted in a Packard model 3002 Auto-Gamma scintillation spectrometer. Counting rates that were no more than 2 standard deviations above replicate blank values were scored as having no activity. The activity in each whole organ was expressed as a percentage of the radioactivity injected. The total activity in the bone marrow was expressed as the activity in one femur multiplied by 10 and that in the blood was calculated as ct/min/ml × 10 (Wang, 1959).

When $^{3}$H-thymidine-labelled cells had been injected, the lung, liver and spleen were removed from recipient rats, fixed in formol saline and subjected to standard histologic and autoradiographic techniques.

### RESULTS

### (i) *Fate of i.v.-injected lymphoid leukaemia cells (HRL) in normal syngeneic rats*

The recovery of activity in 30 hooded rats, killed in groups of 5 at various intervals after the injection of *in vitro* $^{125}$IUDR-labelled HRL cells, is shown in the upper part of Fig. 1. Immediately after injection the highest activity, 36% of that injected, was found in the lung and 9% was recovered from the blood. However, 40% of the blood radioactivity was found to be in the serum, indicating that at least this amount was not attached to the injected cells. The activity recovered in the lung and blood decreased with time, whilst that in the liver increased to a maximum at 2 and 4 h after injection, of about 17% of that injected. The total radioactivity recovered from the lung, liver, spleen and gut fell progressively with time and by 24 h only 7·5% of the total activity injected could be detected in these organs (Fig. 2). The counts in the bone marrow (activity in one femur × 10) were relatively high and increased to a maximum at 2–4 h after injection (Table).

A few rats were killed at 4 and 24 h, totally dissected, and the activity present in the various tissues of the whole animal counted. In the 4-h rats 7·5% of the activity injected was found in the kidney and bladder fraction, indicating a high $^{125}$I excretion; and in the 24-h rats 10% was found in the thyroid, which binds free iodine. There was some radioactivity throughout the other tissues but this was much too low to indicate that there was significant homing to tissues other than those routinely sampled.

Autoradiographic studies of the lung, liver and spleen after the injection of $^{3}$H-labelled HRL cells showed that at 0·5 h after their injection a few cells were present in the peripheral capillaries of the terminal alveoli of the lung, and some cells which appeared damaged were in the liver. At 4 h the liver showed a scattering of label with few intact labelled cells, and in the spleen there were a few labelled cells in the red pulp but none in the white pulp.

The recovery of radioactivity from the viscera of recipients which had received heat-killed labelled HRL cells is also shown in Fig. 2. The loss of injected activity was more rapid from these animals than from those that had received labelled viable cells. The activity that was recovered was found mainly in the lungs and liver. It was notable that more heat-killed cells were found in the lungs (36% at 0·5 h after injection) than labelled viable cells (16%).

An attempt was made to discern how far damage sustained during *in vitro* labelling contributed to the initial trapping in the lung and subsequent lysis of the injected cells. Leukaemia

FIG. 1.—The distribution of radioactivity in the organs of normal and leukaemic hooded rats, as a percentage of the injected dose, at various times after they had received an i.v. injection of $5 \times 10^7$ $^{125}$IUDR-labelled HRL cells. Each histogram represents the mean value from 5 recipients; the bar indicates the range of results.

blast cells were labelled *in vivo* and injected into syngeneic recipients as whole blood. These labelled cells were trapped to a much smaller extent in the lung (4% immediately after injection), than were cells that had been labelled *in vitro* (36%), but the rate of loss of label with time was approximately the same, there being only 12% of the injected activity recoverable 24 h after injection.

(ii) *Fate of i.v.-injected HRL cells in leukaemic syngeneic rats*

*In vitro*-labelled HRL cells were injected into rats which had received $5 \times 10^6$ leukaemia cells 13–14 days earlier and which had $5 \times 10^5$ blast cells/mm$^3$ blood. There was a different distribution of labelled cells in the leukaemic than in the normal rats (Fig. 1 and 2). The initial retention in the lungs was very much less than in normal rats and the level in the blood very much higher. Indeed, the blast cells circulated in the blood for a much longer period in the leukaemic rats, as can be seen in Fig. 3. The level of activity recovered in the

FIG. 2.—The percentage of injected activity recovered from the lung, liver, spleen and gut at various times after the injection of (a) viable $^{125}$IUDR-labelled HRL cells in normal rats —●—●—, (b) heat-killed labelled cells in normal rats —○— —○—, and (c) labelled cells in leukaemic rats —▲— —▲—. Each point represents the mean value from 5 recipients; the bar indicates the range of results.

FIG. 3.—The percentage of injected activity recovered from the blood at various times after the injection of $^{125}$IUDR-labelled HRL and SAL leukaemia cells in normal —▲—▲— and leukaemic rats —▲— —▲—. Each point represents the mean value from 5 recipients; the bar indicates the range of results.

TABLE.—*Activity Recovered in the Bone-marrow (Counts in Femur × 10) Expressed as a Percentage of the Injected Dose, after Injection of $^{125}$IUDR-labelled Lymphoid (HRL) or Myeloid (SAL) Leukaemia Cells into Syngeneic Normal or Leukaemic Rats*

| Treatment of rats | % activity recovered* Time after injection of cells (h) | | | |
|---|---|---|---|---|
| | 0 | 2 | 4 | 24 |
| HRL cells → normal hooded rat | 2 (1–2·5) | 9 (7–11) | 9 (7–10) | 6 (5–7) |
| HRL cells → leukaemic hooded rat | 0·1 (0·1) | 0·5 (0·1–0·6) | 0·5 (0·1–0·7) | 0·1 (0–0·5) |
| SAL cells → normal August rat | 0 | 3·5 (2–5) | 3·5 (3–4) | 2·5 (2–5) |
| SAL cells → leukaemic August rat | 0 | 1·0 (0·5–2) | 1·0 (0·5–2) | 1·0 (0·5–1·5) |

* Mean of 5 rats (and range of values).

FIG. 4.—The distribution of radioactivity in the organs of normal and leukaemic August rats, as a percentage of the injected dose, at various times after they had received an i.v. injection of $5 \times 10^7$ $^{125}$IUDR-labelled SAL cells. Each histogram represents the mean value from 5 recipients; the bar indicates the range of results.

liver and spleen was not significantly different in the leukaemic rats from that in normal animals. However, the activity in the bone marrow was much reduced (Table).

(iii) *Fate of i.v.-injected myeloid leukaemia cells (SAL) in syngeneic rats*

Experiments similar to those with *in vitro*-labelled HRL cells were performed with *in vitro*-labelled SAL cells injected into syngeneic August rats. Figure 4 shows the distribution of activity after the i.v. injection of cells. Immediately after injection, 60% of the activity injected was recovered from the lung. With time, the activity in the lung decreased, whilst that in the liver (14%) and blood (8%) increased to a maximum at 0·5 and 2 h after injection, respectively. Activity recovered from the bone marrow was at a maximum at 2–4 h after injection (Table). Thereafter the activity recovered from these tissues decreased. Radioactivity in the small gut, thymus and lymph nodes was low. In rats totally dissected and counted, no tissues other than those routinely sampled, excepting

FIG. 5.—The percentage of injected activity recovered from the lung, liver, spleen, and gut at various times after the injection of (a) viable $^{125}$IUDR-labelled SAL cells in normal rats —●—●—, (b) heat-killed labelled cells in normal rats —○— —○—, and (c) labelled cells in leukaemic rats —▲— —▲—. Each point represents the mean value from 5 recipients; the bar indicates the range of results.

the bladder and thyroid, were found to be heavily labelled. The decrease in activity recovered from the viscera with time is shown in Fig. 5. There was a more rapid destruction of heat-killed than of viable SAL cells.

(iv) *Fate of i.v.-injected SAL cells in leukaemic syngeneic rats*

When labelled cells were injected into rats with a florid leukaemia, induced by injecting $10^5$ SAL cells 6–7 days previously, initial trapping in the lung was reduced to half and the level in the blood was more than doubled (Fig. 3 and 4). The activity recovered from the bone marrow was only one-third of that found for normal rats (Table). However, the rate at which the cells held in the viscera were lysed was not significantly different in leukaemic rats from that in normal rats (Fig. 5).

### DISCUSSION

The fates of two i.v.-injected leukaemia blast cell populations, lymphoid (HRL) and myeloid (SAL), were determined in normal and leukaemic recipients. The cells were routinely labelled *in vitro* with $^{125}$IUDR. This analogue of thymidine is incorporated exclusively into the DNA of proliferating cells, is released only on cell death and is not re-utilized (Hughes *et al.*, 1964; Commerford, 1965).

The characteristic feature of both of the leukaemias studied was the rapid rate at which the i.v. injected cells were destroyed. By 24 h between 70 and 80% of the injected activity had been totally eliminated from the body (" elimination " including some uptake by the thyroid). Autoradiographs showed that little of the remaining radioactivity was in intact cells and the destruction of the injected cells was even greater than the loss of radioactivity. Within minutes of injection, more than 90% of the cells had left the blood and in the case of the myeloid leukaemia 65% of the injected cells were trapped in the lung. For the lymphatic leukaemia the extent of the immediate sequestration in the lung was less, possibly because the HRL cells were smaller. Over the first 4 h after injection the radioactivity in liver, spleen and bone marrow increased for both SAL and HRL, suggesting that some of the cells that were trapped in the lung succeeded in escaping to these tissues. This movement was more marked with the HRL than the SAL and at 4 h the residual radioactivity in the lung corresponded to 15% for SAL and only 2% for the HRL. While there was a difference in distribution of activity between lung, liver and spleen for the two leukaemias, the rate at which the radioactive material was eliminated from these organs was almost exactly the same (compare Figs. 2 and 5). However, the elimination of radioactivity from the blood was faster for SAL than for HRL (Fig. 3).

These migratory patterns of the two leukaemia blast cells differ profoundly from that of normal immunoblasts obtained from the rat thoracic duct (Hall and Smith, 1970). On i.v. injection, the latter are arrested for a short period in the lungs and then home preferentially to the small gut where at 4 h 20% of the total radioactivity can be recovered. Secondarily, they migrate to lymph nodes and spleen.

The fate of i.v.-injected labelled leukaemic cells was studied in rats whose blood count was high, and which were within 2–3 days of death from leukaemia. The principal effect of the pre-existing leukaemias for both SAL and HRL was to halve the fraction of cells that were trapped in the lung immediately after injection (see Figs. 1 and 4), to reduce the uptake in the bone marrow (Table), and to double the number of cells that remained in the blood (Fig. 3). This suggests that the reactions which result in the immediate trapping in the lung of the injected cells and their later sequestration in the bone marrow were impaired by the disease, which was far advanced. A consequence of the reduced trapping was that the number of leukaemia cells which remained in the circulation was greatly increased and in the leukaemic rats constituted a significant proportion of the total injected material (Fig. 3). A similar observation has been made in man; Stryckmans *et al.* (1968) found that a large number of autotransfused human leukaemia cells continued to circulate in the blood for at least 24–48 h after their injection.

A surprising finding was that the rate at which radioactivity was eliminated from the RE system (*i.e.* lung, spleen and liver) was not significantly different from that in normal rats. If the injected labelled cells truly mimic the behaviour of the unlabelled leukaemia cells in the leukaemic rats, these results suggest that the RE system in such rats destroys leukaemic cells at a very high rate, in spite of the fact that the organs are extensively infiltrated. Taken at face value, the results of the comparison of leukaemic with normal rats imply (1) that, for both SAL and HRL, progress of the leukaemia is associated with a decrease in the initial trapping in the lung of the circulating cells and a reduction in the later sequestration of cells in the bone marrow; these effects may contribute to the very sudden increase in cells in the blood as the disease advances, and (2) that even when the disease is very advanced, the death rate of both HRL and SAL cells within the RE system is very high. However, caution must be exercised in equating the behaviour of injected radio-labelled cells with the leukaemia cells actually within the rats and it is possible that the damage sustained during labelling *in vitro* may have rendered the injected cells more susceptible to control by the RE system.

### REFERENCES

Alexander, P. & Evans, R. (1971) Endotoxin and Double Stranded RNA Render Macrophages Cytotoxic. *Nature, New Biol.*, **232**, 76.

van den Brenk, H. A. S., Burch, W. M., Kelly, H. & Orton, C. (1975) Venous Diversion Trapping and Growth of Blood-borne Cancer Cells En Route to the Lungs. *Br. J. Cancer*, **31**, 46.

Commerford, S. L. (1965) Biological Stability of 5-Iodo-2′-Deoxyuridine Labelled with $^{125}$-Iodine after its Incorporation into Deoxyribonucleic Acid of the Mouse. *Nature, Lond.*, **206**, 949.

Fidler, I. J. (1970) Metastasis: Quantitative Analysis of Distribution and Fate of Tumour Emboli Labelled with $^{125}$I-5-iodo-2′-deoxyuridine. *J. natn. Cancer Inst.*, **45**, 775.

Fidler, I. J. (1973) The Relationship of Embolic Homogeneity, Number, Size, and Viability to the Incidence of Experimental Metastasis. *Eur. J. Cancer*, **9**, 223.

Hall, J. G. & Smith, M. E. (1970) Homing of Lymph-borne Immunoblasts to the Gut. *Nature, Lond.*, **226**, 262.

Hibbs, J. B., Lambert, L. H. & Remington, J. S. (1972) Non-immunological Destruction of Cells with Abnormal Growth Characteristics by Activated Macrophages. *Proc. Soc. exp. Biol. Med.*, **139**, 1049.

Hofer, K. G., Prensky, W. & Hughes, W. L. (1969) Death and Metastatic Distribution of Tumour Cells in Mice Monitored with $^{125}$I-Iododeoxyuridine. *J. natn. Cancer Inst.*, **43**, 763.

HUGHES, W. L., COMMERFORD, S. L., GITLIN, D., KRUEGER, R. C., SCHULTZE, B., SHAH, V. & REILLY, P. (1964) Deoxyribonucleic Acid Metabolism *In vivo*: I. Cell Proliferation and Death as Measured by Incorporation and Elimination of Iododeoxyuridine. *Fed. Proc.*, **23**, 640.

PARR, I., WHEELER, E. & ALEXANDER, P. (1973) Similarities of the Anti-tumour Actions of Endotoxin, Lipid A and Double-stranded RNA. *Br. J. Cancer*, **27**, 370.

PROCTOR, J., RUDENSTAM, C. M. & ALEXANDER, P. (1973) Increased Incidence of Lung Metastases Following Treatment of Rats Bearing Hepatomas with Irradiated Tumour Cells and the Beneficial Effect of *Corynebacterium parvum* in this system. *Biomedicine*, **19**, 248.

VAN PUTTEN, L. M., KRAM, L. K. J., VAN DIERENDONCK, H. H. C., SMINK, T. & FÜZY, M. (1975) Enhancement by Drugs of Metastatic Lung Nodule Formation after Intravenous Tumour Cell Injection. *Int. J. Cancer*, **15**, 588.

SCHNEIDER, W. C. (1945) Phosphorus Compounds in Animal Tissues. I. Extraction and Estimation of Deoxypentose Nucleic Acid and of Pentose Nucleic Acid. *J. biol. Chem.*, **161**, 293.

SELECKI, E. E. (1959) A Study of the Metastatic Distribution of Ehrlich Ascites Tumour Cells in Mice. *Aust. J. exp. Biol. Med.*, **37**, 489.

SHORTER, R. G. & BOLLMAN, J. L. (1960) Experimental Transfusion of Lymphocytes. *Am. J. Physiol.*, **198**, 1014.

STRYCKMANS, P. A., CHANANA, A. D., CRONKITE, E. P., GREENBERG, M. L. & SCHIFFER, L. M. (1968) Studies on Lymphocytes. *Eur. J. Cancer*, **4**, 241.

WANG, L. (1959) Plasma Volume, Cell Volume, Total Blood Volume and F Cells Factor in the Normal and Splenectomized Sherman Rat. *Am. J. Physiol.*, **196**, 188.

WEISS, L., GLAVES, D. & WAITE, D. A. (1974) The Influence of Host Immunity on the Arrest of Circulating Cancer Cells, and its Modification by Neuraminidase. *Int. J. Cancer*, **13**, 850.

WOODRUFF, J. J. & GESNER, B. M. (1969) The Effect of Neuraminidase on the Fate of Transfused Lymphocytes. *J. exp. Med.*, **129**, 551.

WRATHMELL, A. B. (1976) The Growth Patterns of Two Transplantable Acute Leukaemias of Spontaneous Origin in Rats. *Br. J. Cancer*, **33**, 172.

WRATHMELL, B. & ALEXANDER, P. (1973) Growth Characteristics and Immunological Properties of a Myeloblastic and a Lymphoblastic Leukaemia in Pure Line Rats. In: *V. Immunologic Aspects of Leukemia: Unifying Concepts of Leukemia.* Ed. R. M. Dutcher and L. Chieco-Bianchi. *Bibl. haemat.*, **39**, 649.


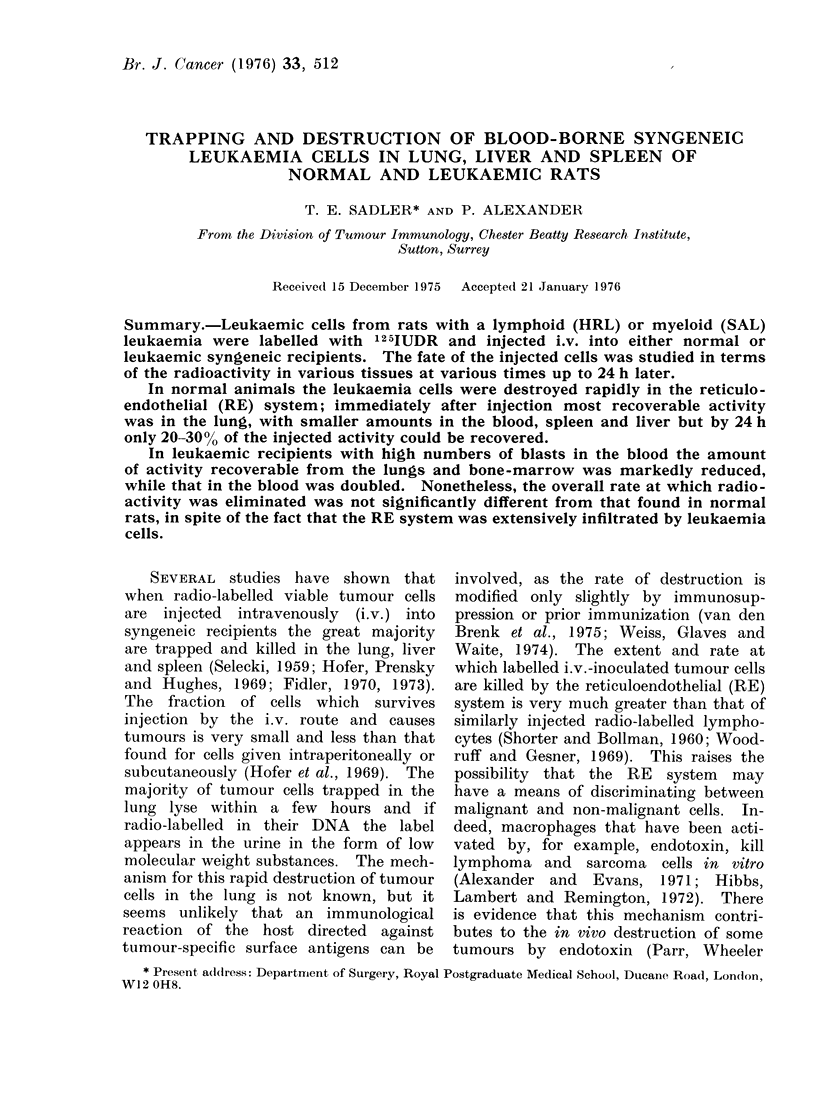

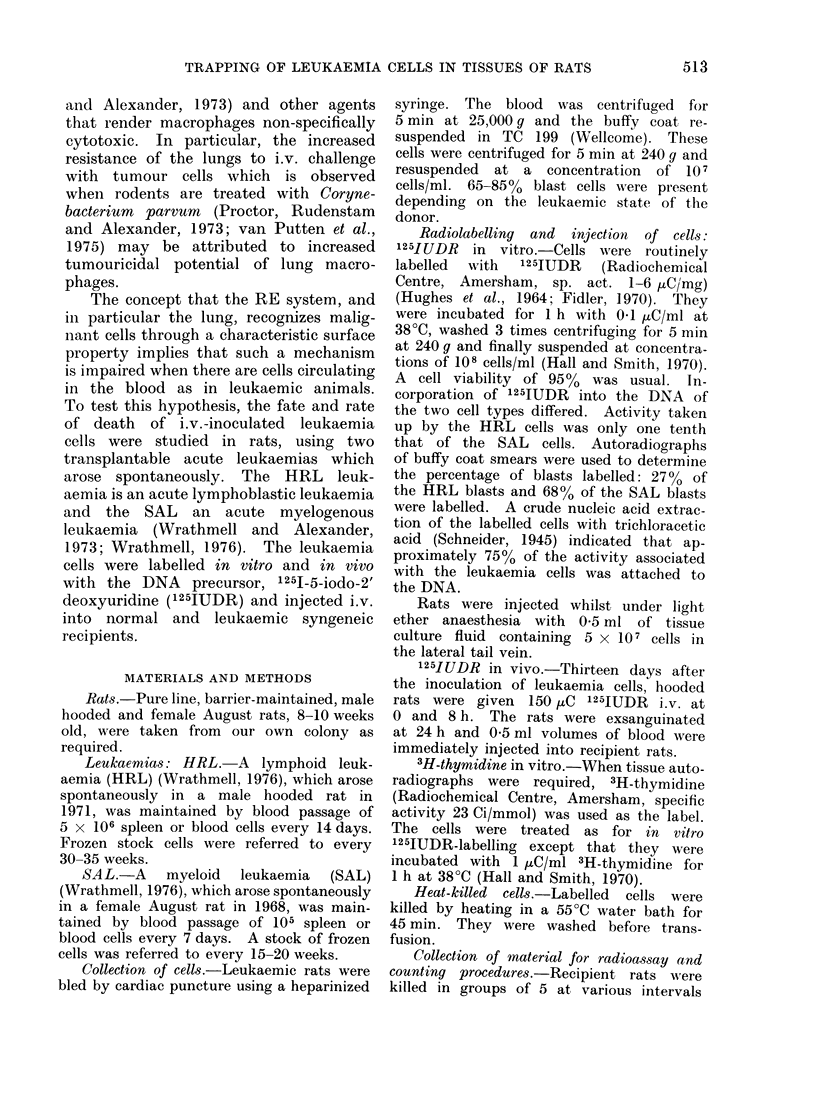

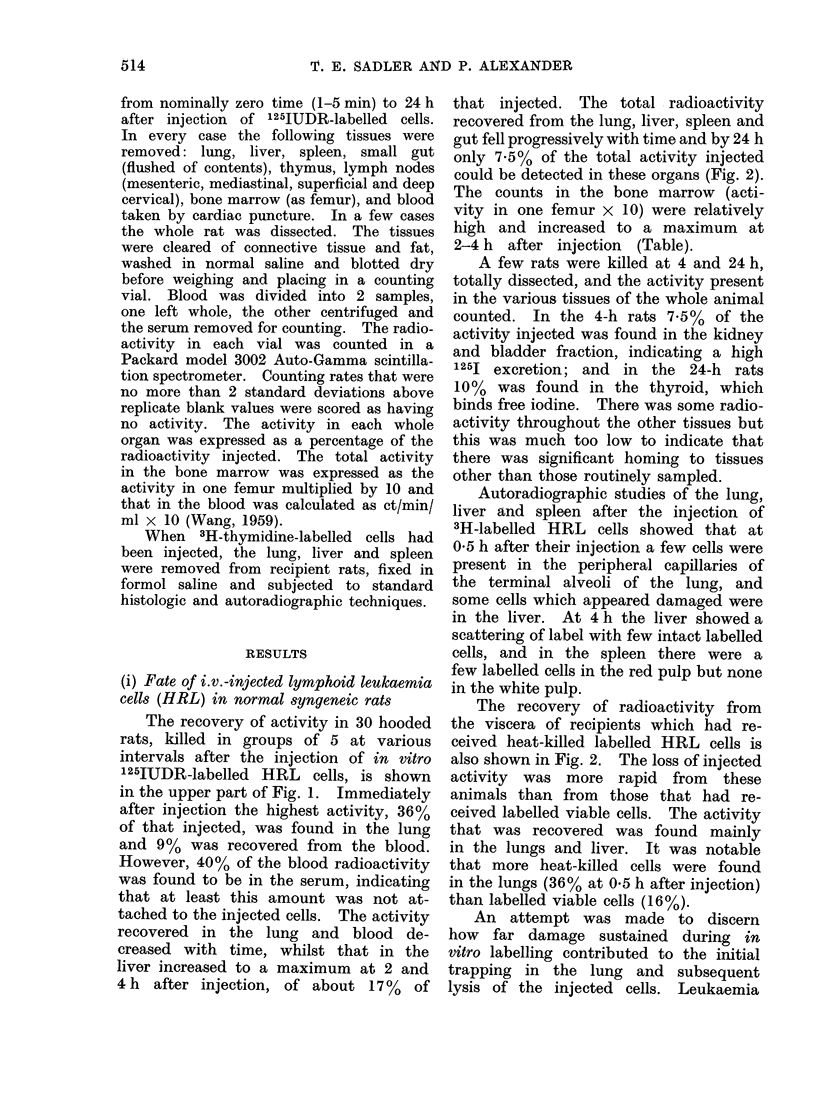

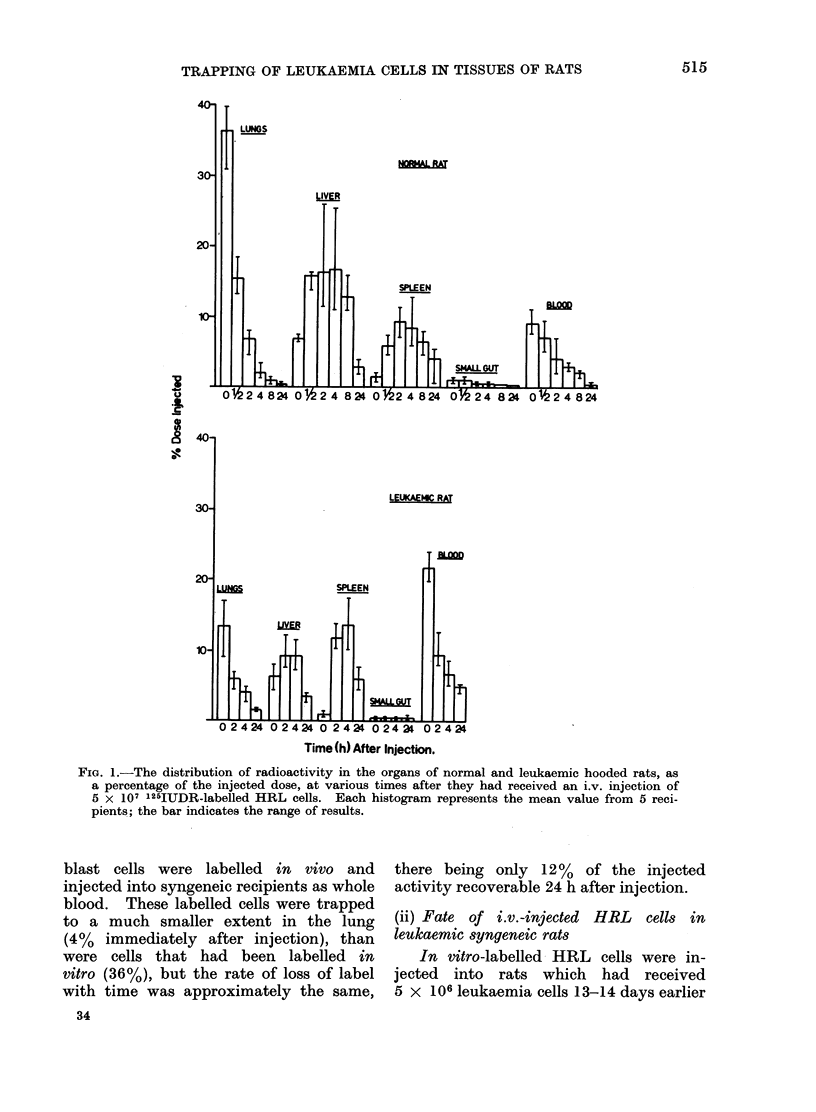

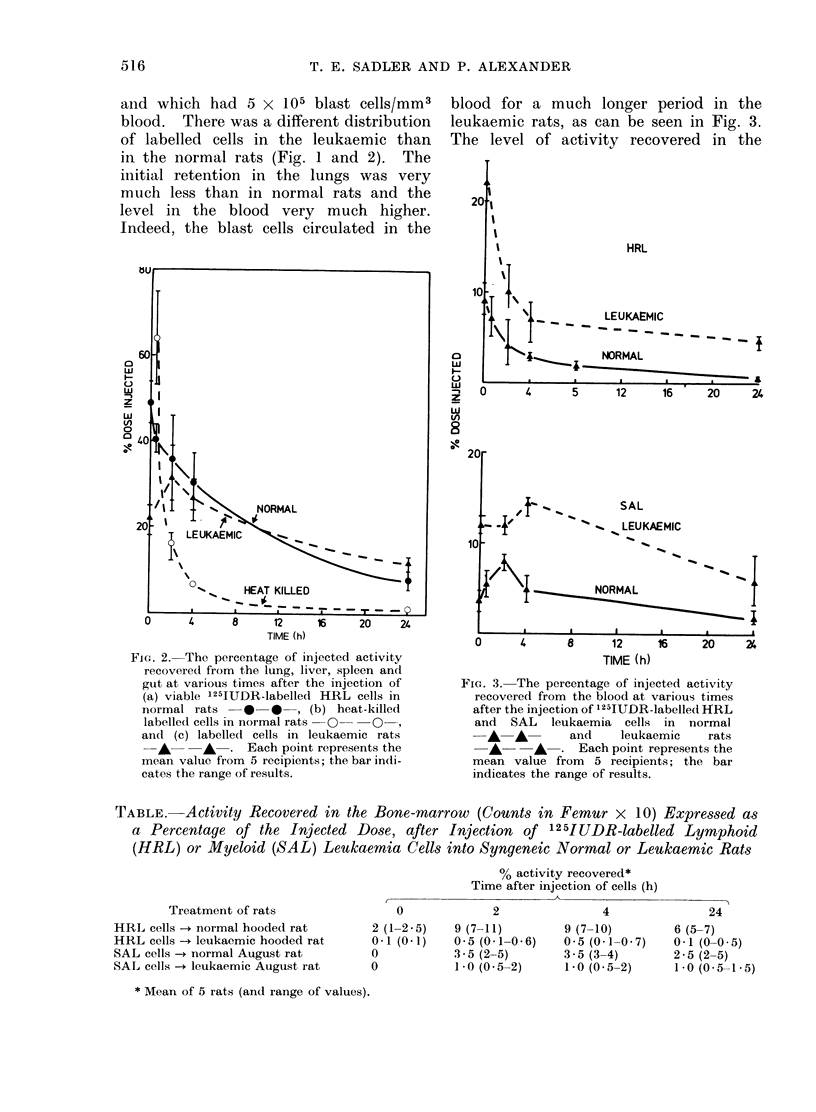

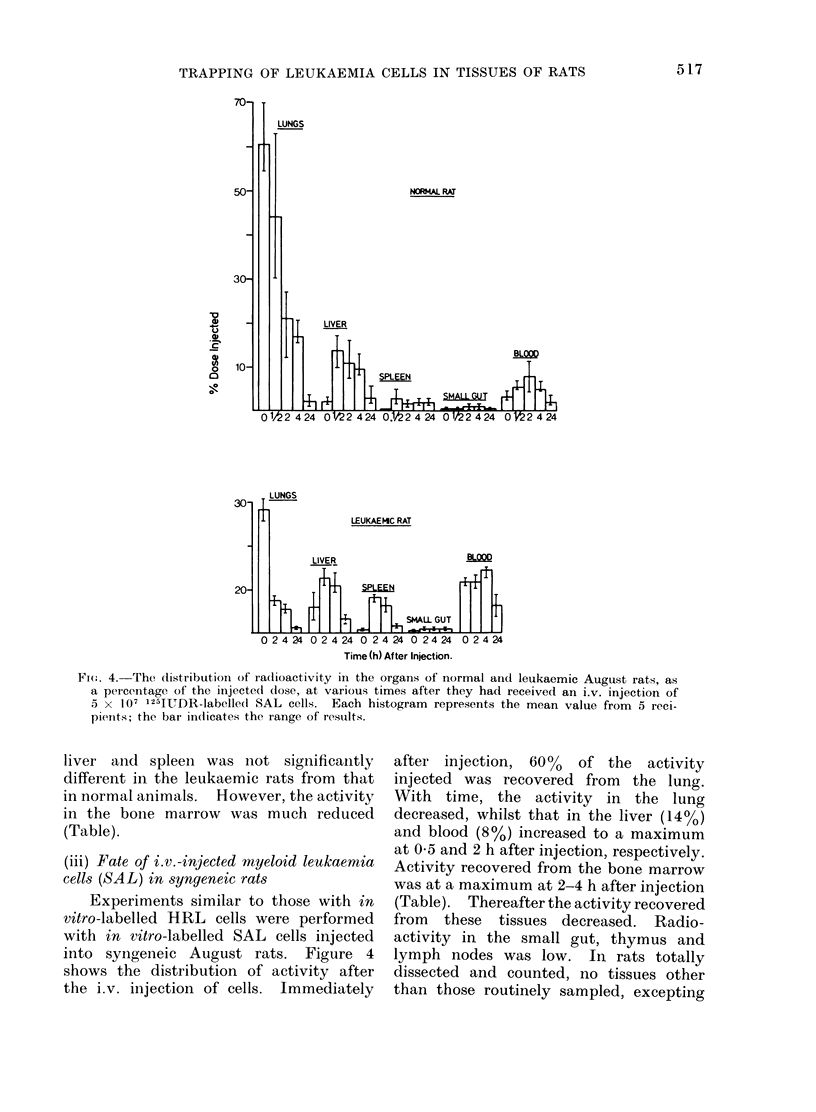

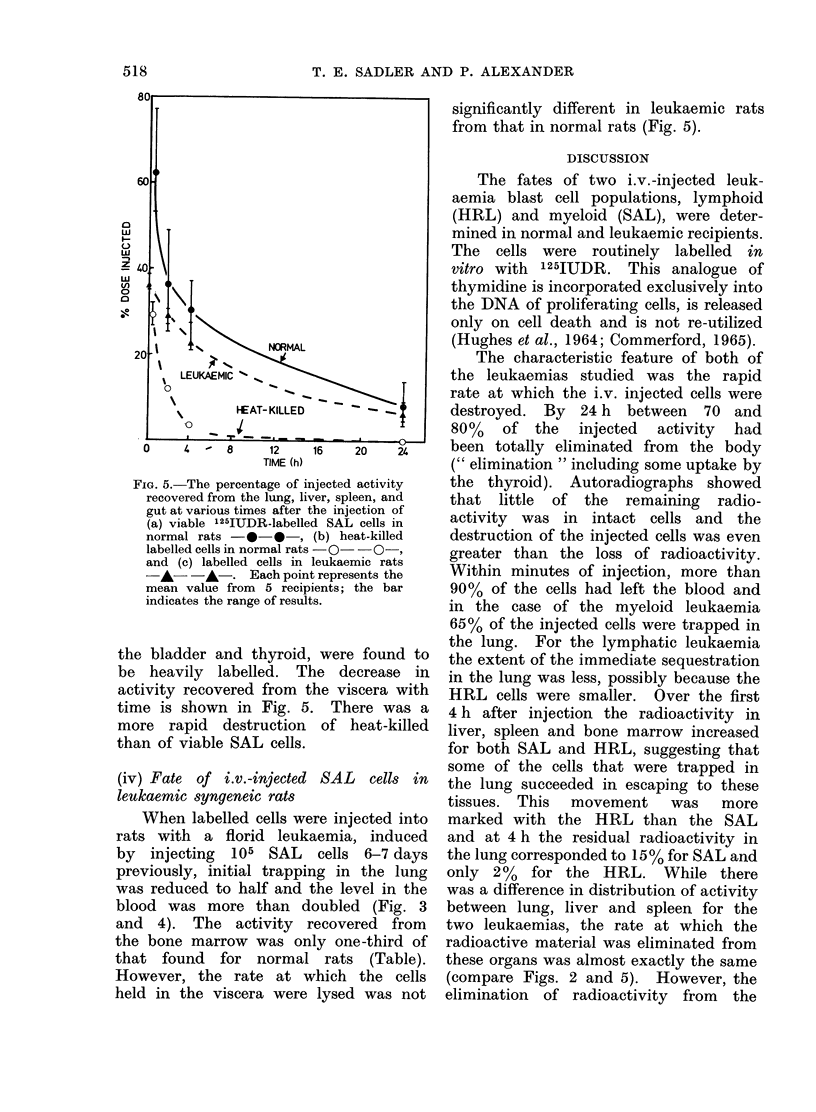

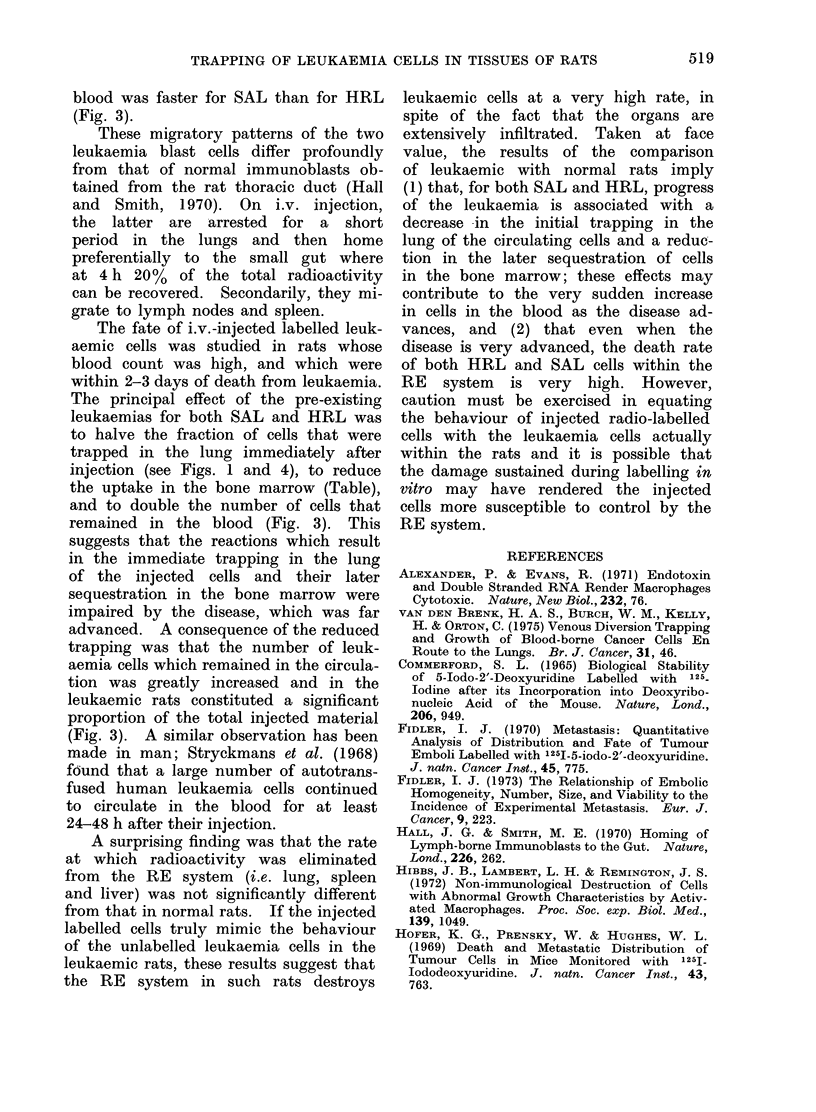

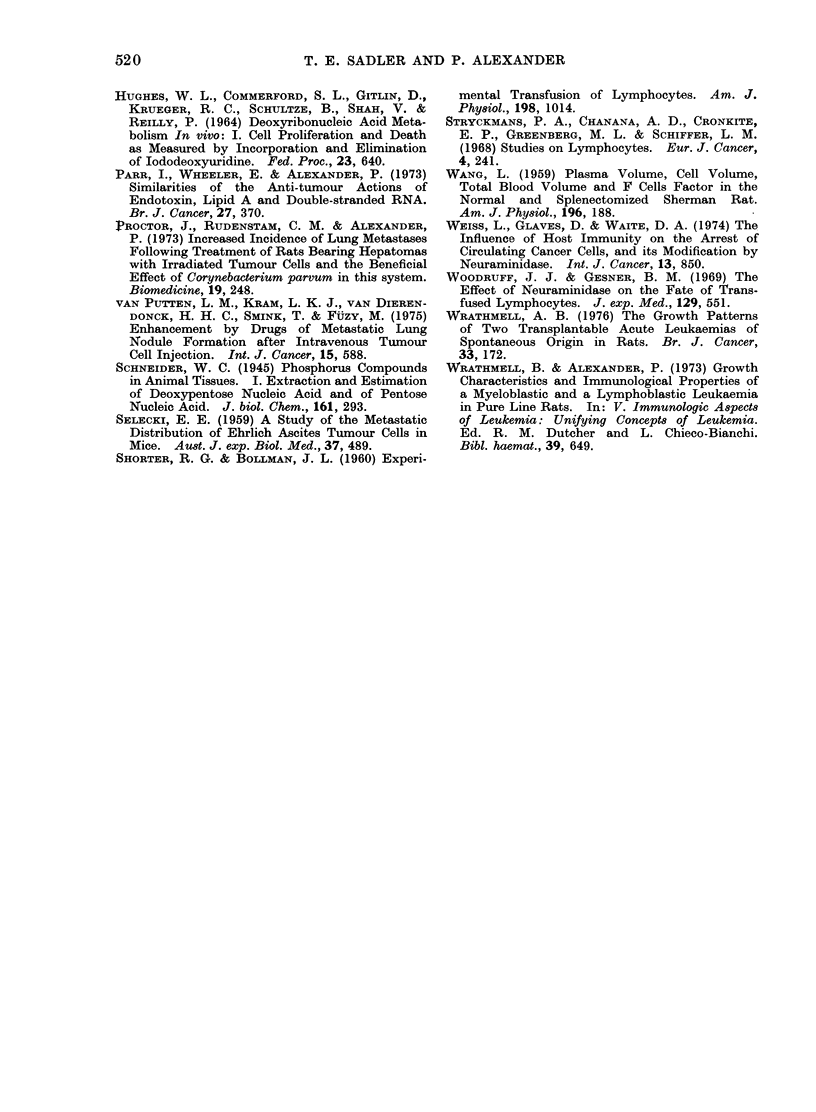


## References

[OCR_00926] Alexander P., Evans R. (1971). Endotoxin and double stranded RNA render macrophages cytotoxic.. Nat New Biol.

[OCR_00937] Commerford S. L. (1965). Biological stability of 5-iodo-2'-deoxyuridine labelled with iodine-125 after its incorporation into the deoxyribonucleic acid of the mouse.. Nature.

[OCR_00950] Fidler I. J. (1973). The relationship of embolic homogeneity, number, size and viability to the incidence of experimental metastasis.. Eur J Cancer.

[OCR_00977] HUGHES W. L., COMMERFORD S. L., GITLIN D., KRUEGER R. C., SCHULTZE B., SHAH V., REILLY P. (1964). DEOXYRIBONUCLEIC ACID METABOLISM IN VIVO: I. CELL PROLIFERATION AND DEATH AS MEASURED BY INCORPORATION AND ELIMINATION OF IODODEOXYURIDINE.. Fed Proc.

[OCR_00956] Hall J. G., Smith M. E. (1970). Homing of lymph-borne immunoblasts to the gut.. Nature.

[OCR_00961] Hibbs J. B., Lambert L. H., Remington J. S. (1972). In vitro nonimmunologic destruction of cells with abnormal growth characteristics by adjuvant activated macrophages.. Proc Soc Exp Biol Med.

[OCR_00968] Hofer K. G., Prensky W., Hughes W. L. (1969). Death and metastatic distribution of tumor cells in mice monitored with 125I-iododeoxy-uridine.. J Natl Cancer Inst.

[OCR_00985] Parr I., Wheeler E., Alexander P. (1973). Similarities of the anti-tumour actions of endotoxin, lipid A and double-stranded RNA.. Br J Cancer.

[OCR_00991] Proctor J., Rudenstam C. M., Alexander P. (1973). Increased incidence of lung metastases following treatment of rats bearing hepatomas with irradiated tumour cells and the benefical effect of Corynebacterium parvum in this system.. Biomedicine.

[OCR_01012] SELECKI E. E. (1959). A study of the metastatic distribution of Erlich ascites tumour cells in mice.. Aust J Exp Biol Med Sci.

[OCR_01017] SHORTER R. G., BOLLMAN J. L. (1960). Experimental transfusion of lymphocytes.. Am J Physiol.

[OCR_01022] Stryckmans P. A., Chanana A. D., Cronkite E. P., Greenberg M. L., Schiffer L. M. (1968). Studies on lymphocytes. IX. The survival of atuotransfused labeled lymphocytes in chronic lymphocytic leukemia.. Eur J Cancer.

[OCR_00931] Van Den Brenk H. A., Burch W. M., Kelly H., Orton C. (1975). Venous diversion trapping and growth of blood-borne cancer cells en route to the lungs.. Br J Cancer.

[OCR_01028] WANG L. (1959). Plasma volume, cell volume, total blood volume and F cells factor in the normal and splenectomized Sherman rat.. Am J Physiol.

[OCR_01034] Weiss L., Glaves D., Waite D. A. (1974). The influence of host immunity on the arrest of circulating cancer cells, and its modification by neuraminidase.. Int J Cancer.

[OCR_01040] Woodruff J. J., Gesner B. M. (1969). The effect of neuraminidase on the fate of transfused lymphocytes.. J Exp Med.

[OCR_01045] Wrathmell A. B. (1976). The growth patterns of two transplantable acute leukaemias of spontaneous origin in rats.. Br J Cancer.

[OCR_01051] Wrathmell A., Alexander P. (1973). Growth characteristics and immunological properties of a myeloblastic and a lymphoblastic leukaemia in pure line rats.. Bibl Haematol.

[OCR_01001] van Putten L. M., Kram L. K., van Dierendonck H. H., Smink T., Füzy M. (1975). Enhancement by drugs of metastatic lung nodule formation after intravenous tumour cell injection.. Int J Cancer.

